# Challenging the Portrait of the Unhealthy Gamer—The Fitness and Health Status of Esports Players and Their Peers: Comparative Cross-Sectional Study

**DOI:** 10.2196/45063

**Published:** 2023-08-03

**Authors:** Sascha Ketelhut, Alex Bodman, Thomas Ries, Claudio R Nigg

**Affiliations:** 1 Department of Health Sience Institute of Sport Science University of Bern Bern Switzerland; 2 German Sport University Cologne Cologne Germany

**Keywords:** blood pressure, gamers, health status, physical activity behavior, physical fitness, esports, health burden, sedentary athlete, health, fitness

## Abstract

**Background:**

Esports players are often referred to as sedentary athletes, as gaming requires prolonged sedentary screen exposure. As sedentary behavior and physical inactivity are major causes of noncommunicable diseases and premature death, esports players may be at an increased risk for health implications. Prior research has established esports players as having higher levels of body fat and lower levels of lean body mass versus age-matched controls, suggesting the need to assess further health and fitness outcomes of this demographic. However, while research interest is undoubtedly increasing, the majority of studies has focused on subjective self-report data and has lacked relevant objective health and fitness measurements.

**Objective:**

This study aimed to assess the health and fitness status of a group of competitive esports players in relation to an age- and sex-matched comparison group.

**Methods:**

In total, 51 competitive esports players (mean 23, SD 3 years, 2 female) and 51 nonesports players (mean 24, SD 3 years, 2 female) were enrolled in this cross-sectional laboratory study. The esports players and the nonesports players completed a questionnaire assessing demographic data and self-reported physical activity levels. Furthermore, physical parameters including BMI, waist-to-height ratio, body fat percentage, systolic blood pressure, diastolic blood pressure, pulse wave velocity, maximal grip strength, and maximal oxygen consumption were assessed.

**Results:**

There were no significant differences in BMI (*t*_100_=1.54; *P*=.13; *d*=0.30), waist-to-height ratio (*t*_100_=1.44; *P*=.16; *d*=0.28), body fat percentage (*t*_100_=−0.48; *P*=.63; *d*=−0.09), systolic blood pressure (*t*_100_=−0.06; *P*=.93; *d*=−0.01), diastolic blood pressure (*t*_100_=0.37; *P*=.71; *d*=0.07), pulse wave velocity (*t*_93_=−2.08; *P*=.15; *d*=−0.43), maximal grip strength (*t*_100_=−.08; *P*=.94; *d*=−0.02), maximal oxygen consumption (*t*_100_=−0.11; *P*=.92; *d*=−0.02), and physical activity (PA) levels (*t*_86_=2.17; *P*=.08; *d*=0.46) between the groups.

**Conclusions:**

While the health narrative directed toward esports players has been mainly negative, this laboratory-based study indicated that esports players are not less healthy or fit compared to their peers. However, it seems that esports players are very heterogeneous and seem to span across the whole range of the fitness and health spectrum. Thus, the generalized statements of the esports athlete as an obese and unhealthy individual may need to be reconsidered.

## Introduction

The rapid expansion of esports (competitive video gaming) viewership and subsequent participation, combined with the inherent sedentary nature of esports, has raised concern for the overall health of these players [1,2]. Multiple studies have shown a positive correlation between video game play time and sedentary behavior [3,4]. Gaming sessions, especially during periods of competition and training, can typically span 3-6 hours of uninterrupted sitting [5].

Increased sitting time is associated with a higher risk of cardiovascular disease (CVD), obesity, and diabetes [6,7]. The sedentary nature of esports may predispose players to multiple adverse health outcomes due to the growing evidence identifying sedentary behavior as a potential risk factor independent of physical activity (PA) levels [8,9]. Prior research has established esports players as having higher levels of body fat and lower levels of lean body mass versus age-matched controls, suggesting the need to assess further health and fitness outcomes of this demographic [10]. Research has only now begun gaining traction on the topic of esports player health and has established numerous potential health risk implications among these players such as physical inactivity, high levels of sedentary behavior, stress, sleep problems, musculoskeletal pain, overuse injuries, and metabolic disorders [5,11,12].

However, the current literature on the health of esports players has produced conflicting results. For example, a study conducted by Rudolf et al [13] on 1066 esports players revealed that 95% of respondents reported having good to excellent health status, and nearly two-thirds of them achieved the recommended levels of PA. Similarly, Giakoni-Ramírez et al [14] found that more than 90% of the professional esports players in their sample achieved high or moderate levels of PA. Furthermore, professional esports players have been found to integrate approximately 1.08 hours of physical exercise into their daily training, as a strategy to improve gameplay, manage stress, and promote general health [15]. Especially high level esports players have been reported to have a lower BMI and reach higher PA levels [16,17].

The existing research on the health of esports players reveals significant heterogeneity among this population. Therefore, further investigation is needed to better evaluate the potential health risks associated with esports.

While research interest is undoubtedly increasing, the majority of studies has focused on subjective self-report data and has lacked relevant objective health and fitness measurements [18]. No previous study has assessed maximal oxygen consumption (VO_2_max) and different hemodynamic parameters like blood pressure (BP) or pulse wave velocity (PWV), which are arguably among the strongest predictors of future health, all-cause mortality, and cardiovascular risks [19-23].

Establishing if competitive esports players have poor health and fitness outcomes compared to peers of a similar sex and age would incentivize the implementation of health promotion strategies in a field currently lacking any type of structured health education or fitness training protocols [5,15,24]. Unfortunately, there is inconclusive evidence among the current literature on the overall health and fitness status of competitive esports players. Moreover, there is a lack of objective fitness and health assessments among this population. Thus this study aims to objectively assess competitive esports player health and fitness in relation to an age- and sex-matched comparison group. The results should help to determine if the unhealthy and unfit narrative surrounding these players is warranted and targeted health promotion efforts are needed.

## Methods

### Participants and Study Design

A cross-sectional study design was implemented involving a sample of competitive esports players recruited from individuals living in Switzerland to assess overall health and fitness status. A sample consisting of nonesports playing individuals, also living in Switzerland, was recruited and assessed to establish an age- and sex-matched comparison group for comparison. Recruitment was conducted at different Universities and Universities of Applied Sciences throughout Switzerland. Recruitment and data collection were conducted between May 2021 and September 2022.

Esports participants were eligible for the study if they (1) were active members of an esports club, or played video or computer games competitively (participating in organized competitions, tournaments, and web-based matches with the goal of winning and improving their skills), (2) were between the ages of 16 and 45 years, (3) had no physical limitations to exercise, (4) provided written informed consent, and (5) were not taking antihypertensive or other cardiovascular medications. The same was true for the comparison group participants, however, they were not allowed to be active members of an esports club or play video or computer games regularly (<3 hours per week) or competitively.

A total of 102 participants took part in the study. The sample included 51 esports players and 51 nonesports players in the comparison group. There was no difference in mean age (*P*=.09) between the esports players (mean 23, SD 3 years) and the comparison group (mean 24, SD 3 years). Both groups had 2 female participants.

### Ethics Approval

The experimental procedures of the study were approved by the Ethical Commission of the Faculty of Human Sciences University of Bern (2021-02-00005). The participants received a verbal and written explanation of the study’s objective and assessment procedures and provided written informed consent.

### Procedure

The study was conducted at the Institute of Sports Science of the University of Bern. All measurements were carried out by the principal investigator under controlled conditions using the same equipment and procedure.

Participants were instructed prior to their assessment day to have completed their last meal and refrain from consuming sugar-sweetened beverages 2 hours prior to the assessment. Participants were also instructed to refrain from consuming caffeinated, alcoholic beverages or nicotine 4 hours before the assessment and to avoid any intense PA for 48 hours before the assessment.

All measurements were conducted on the same assessment day for each respective participant. All participants signed a physical activity readiness questionnaire. Participants then sat in a quiet room and filled out a questionnaire that included demographic questions and a physical activity questionnaire [25]. Participants could complete the survey in either German or English, depending on their preference. Thereafter, different physical measurements were obtained in the following order: height, body mass, body fat, waist circumference, BP, maximal grip strength (MGS), and cardiorespiratory fitness.

### Measurements

#### Anthropometrics

Anthropometric measurements were taken with no shoes (height, body mass, and waist circumference). The waist circumference was measured midway between the lowest ribs and the iliac crest to the nearest 0.5 cm. A bioimpedance scale (Tanita RD-545, Tanita Europe BV) was used to assess weight and calculate body fat percentage (BF%). BMI was calculated as a function of weight in kilograms and height in meters (kg/m^2^), and waist-to-height ratio (WHtR) was calculated as a function of waist circumference and height (waist circumference/height).

#### Hemodynamics

Resting systolic blood pressure (SBP) and diastolic BP, as well as PWV, were measured using the Mobil-O-Graph (24 PWA monitor, IEM), which is a clinically validated device for hemodynamic measurements [26]. Measurements were obtained after a 10-min resting phase in the supine position. A minimum of 2 readings were taken from the left arm using custom-fit arm cuffs. The arm was placed on an armrest to ensure that the heart and pressure cuff were at the same level.

#### Maximal Grip Strength

A MGS test using a hand dynamometer (Saehan DHD-1, Saehan Corp) was performed by each participant following the standardized positioning recommendations from the American Society of Hand Therapists [27]. This protocol required participants to be seated with shoulders adducted and neutrally rotated and the elbow flexed at 90° with the forearm in a neutral alignment. Wrist position was permitted between 0° and 30° of dorsiflexion. The grip width of the hand dynamometer handle was adjusted to an appropriate width. The hand was required to be positioned so that the thumb was around 1 side of the handle and the other 4 fingers around the opposite side. Participants were then instructed to squeeze as hard as possible for as long as possible until a maximum value in kilograms was recorded on the dynamometer. Participants then performed the test on the alternate side and then had a 2-minute rest period before repeating the test again on both sides. From the 4 total trials, the maximum value recorded was taken for analysis.

#### Physical Fitness

Physical fitness, determined by VO_2_max, was assessed by a graded exercise test on a bicycle ergometer (Ergometrics 800s, Ergoline GmbH). The test started at 50 or 75 W (depending on lean body mass and training status) [28] with a stepwise increment of 25 W/minute. Participants performed a 5-minute warm-up at the respective starting watt level before proceeding to the stepwise performance test. Participants rode on the bicycle until voluntary exhaustion or until a cadence of greater than 60 revolutions per minute could no longer be maintained. To determine whether VO_2_max was attained, 3 of the following five criteria had to be met: (1) a final rating of perceived exertion score of ≥17 on the Borg scale (scale 6-20) [29], (2) a respiratory exchange ratio >1.1, (3) no change in HR with a change in workload, (4) a “plateau” (an increase of no >150 mL) in oxygen uptake with an increase in workload, and (5) volitional fatigue, defined as an inability to maintain a pedal rate above 60 rpm.

Throughout the test, oxygen consumption was collected and analyzed using a breath-by-breath gas collection system (Metalyzer 3B, Cortex). VO_2_max was calculated as the highest recorded value, using the recorded rolling average of 15-second epochs. A 2-point calibration procedure was conducted according to the manufacturer’s guidelines prior to each testing session. The calibration of the oxygen and carbon dioxide sensors was performed with gases of known concentrations. The flow rate was calibrated with a 2-L syringe. In addition, ambient air measurements were conducted before each test.

#### Physical Activity Behavior

Self-report PA was assessed with an adapted version of the Godin Leisure Time Physical Activity Questionnaire (GLTPAQ) [30]. This psychometrically robust measure assesses the frequency of mild, moderate, and strenuous exercise and has been shown to be valid and reliable [30-32]. Apart from the original version, a German adaptation [33] was used. From this questionnaire, the Godin score [30] and weekly minutes per week of moderate to vigorous physical activity (MVPA) were calculated.

### Statistical Analysis

An a priori power analysis utilizing G*power (version 3.1.2; Heinrich Heine Universität), indicated that a sample size of 51 participants in each group would provide sufficient power (80%) to observe differences, assuming a medium effect size. All statistical analyses (available cases) were performed using SPSS Statistics (version 27.0; IBM Corp). The results are presented as mean (SD) values. Data normality was assessed on each variable using a histogram and the Kolmogorov-Smirnoff test. Independent *t* tests were conducted to compare outcomes. Statistical significance was set at *P*<.05. To determine effect size, Cohen *d* was calculated (*d*>.2: small effect; >.5: medium effect; >.8: large effect) [34].

## Results

### Overview

The vast majority (n=46, 90%) of the esports group reported being amateur competitive esports players, not earning any substantial income from competing. Only 5 participants (10%) were semiprofessional players and reported earning a share of their main income from esports (Table 1).

The most popular esports genre was multiplayer web-based battle arenas (n=23, 45%), followed by games from the sports genre (n=12, 24%), and first-person shooters (n=6, 12%). Their average hours playing esports per week was 14 (SD 8). None of the comparison group participants were actively competing in any esports or regularly engaged in any video gaming (Table 1).

**Table 1:** Esports specific data.

		Value
**Esport genre, n (%)**		
	Multiplayer web-based battle arena	23 (45)
	Sports	12 (24)
	First-person shooter	6 (12)
	Sports + first-person shooter	5 (10)
	Sports + multiplayer web-based battle arena	4 (8)
	First-person shooter + multiplayer web-based battle arena	1 (2)
**Level, n (%)**		
	Amateur^a^	45 (88)
	Semiprofessional^b^	6 (12)
Hours of playing (hours per week), mean (SD)		14.02 (7.7)

^a^Not earning any substantial income from esports.

^b^Earning a share of their main income from esports.

### Anthropometrics

There were no significant differences between the esports players and the comparison group in BMI (mean 23.9, SD 3.1 kg/m^2^ vs mean 23.1, SD 2.2 kg/m^2^; *t*_100_=1.54; *P*=.13; *d*=0.30), BF% (mean 18%, SD 6% vs mean 19%, SD 5%; *t*_100_=−0.48; *P*=.63; *d*=−0.09), and WHtR (mean 0.46, SD 0.05 vs mean 0.44, SD 0.04; *t*_100_=1.44; *P*=.16; *d*=0.28; Table 2).

Based on the BMI, 16 (31%) of the esports players were classified as overweight and 2 (4%) as obese [35]. In the age-matched control group, 12 (24%) of the participants were classified as overweight and none as obese. Regarding WHtR, 8 (16%) of the esports players and 4 (8%) of the comparison group were above the established 0.5 health risk threshold for WHtR [36]. Based on BF%, 5 (10%) participants in both groups had values above the obesity thresholds [37].

**Table 2:** Differences in outcomes between esports players and comparison group.

Outcome	Esports players, mean (SD)	Comparison group, mean (SD)	*P* value^a^
Age (years)	23 (3)	24 (3)	.09
Height (m)	1.79 (0.7)	1.80 (0.06)	.16
Body mass (kg)	76.6 (12.0)	75.1 (9.3)	.50
BMI (kg/m^2^)	23.9 (3.1)	23.1 (2.2)	.13
WHtR^b^	0.46 (0.05)	0.44 (0.04)	.16
BF^c^ (%)	18 (6)	19 (5)	.63
SBP^d^ (mm Hg)	121 (8)	121 (7)	.93
DBP^e^ (mm Hg)	73 (7)	72 (7)	.71
PWV^f^ (m/s)	5.2 (0.3)	5.2 (0.4)	.15
VO_2_max^g^ (mL/min/kg)	46 (9)	46 (6)	.92
MGS^h^ (kg)	46 (8)	46 (6)	.94
MVPA^i^ (min)	338 (283)	230 (180)	.08
GLTPAQ score^j^	55 (25)	54 (19)	.80

^a^*P* values indicate difference between esports players and comparison group.

^b^WHtR: waist-to-height ratio.

^c^BF: body fat.

^d^SBP: systolic blood pressure.

^e^DBP: diastolic blood pressure.

^f^PWV: pulse wave velocity.

^g^VO_2_max: maximal oxygen consumption.

^h^MGS: maximal grip strength.

^i^MVPA: moderate to vigorous physical activity.

^j^GLTPAQ score: Godin Leisure Time Physical Activity Questionnaire score.

### Hemodynamics

No difference in SBP (mean 121, SD 8 mm Hg vs mean 121, SD 7 mm Hg; *t*_100_=−0.06; *P*=.93; *d*=−0.01) and diastolic blood pressure (mean 73, SD 7 mm Hg vs mean 72, SD 7 mm Hg; *t*_100_=0.37; *P*=.71; *d*=0.07), as well as PWV (mean 5.2, SD 0.3 mm Hg vs mean 5.2, SD 0.4 mm Hg; *t*_93_=−2.08; *P*=.15; *d*=−0.43) were found (Table 2). According to reference values [38], 5 (10%) of the esports participants and 4 (8%) of the comparison group had a high normal BP. In both groups, only 1 participant revealed BP in the hypertensive range. All PWV values aligned with reference values [39].

### Physical Fitness

Both esports players and comparison group revealed similar VO_2_max (mean 45.9, SD 8.7 mL/min/kg vs mean 46.1, SD 5.7 mL/min/kg; *t*_100_=−0.11; *P*=.92; *d*=−0.02), and MGS (mean 45.9, SD 8.0 kg vs mean 46.0, SD 6.1 kg; *t*_100_=−0.08; *P*=.94; *d*=−0.02; Table 2).

In the esports group, 17 (33%) had excellent to superior VO_2_max scores. In total, 30 (59%) were classified as fair or good, and 4 (8%) as poor or very poor. In the comparison group, 41 (80%) of participants were classified as fair or good, 2 (4%) as very poor or poor, and 8 (16%) as excellent or superior [40].

### Physical Activity Levels

Regarding self-reported physical activity, no significant differences in minutes of MVPA could be detected between the esports players and the comparison group (mean 338, SD 283 minutes vs mean 230, SD 180 minutes; *t*_86_=2.17; *P*=.08; *d*=0.46), and both groups scored similarly on the GLTPAQ (mean 55, SD 25 vs mean 54, SD 19; *t*_86_=0.26; *P*=.80; *d*=0.06; Table 2). According to minutes of MVPA, 42 (82%) of the esports group and 40 (78%) of the comparison group met or exceeded the World Health Organization’s PA guidelines [41].

## Discussion

### Principal Findings

This study aimed to assess the health and fitness status of a sample of competitive esports players in comparison to an age-sex-matched comparison group to determine if esports players are less healthy than their peers. The results showed that this was not the case, as esports players had similar health and fitness results across the board compared to the comparison group. However, while the esports players exhibited a higher percentage of reaching excellent VO_2_max scores, they also showed a greater prevalence of being overweight or obese.

### Anthropometrics

Regarding BMI, WHtR, and BF%, no significant differences could be detected between the groups. Even though esports players showed no significant differences in BMI compared to the comparison group, the percentage of individuals with overweight and those with obesity was higher. Thus, it seems that esports players can be found more at the extreme ends of the BMI continuum. Interestingly, according to BF%, no differences were detected between groups regarding the percentage surpassing the obesity threshold. Therefore, the overweight or obese stereotype of video game players, as is often reported in previous literature cannot be totally confirmed.

The Federal Statistics Office of Switzerland [42] last reported that 20.3% of males aged 15-24 years were overweight, while 5.1% were obese, whereas males aged 25-34 years had 33.7% classified as overweight and 9.2% as obese. The younger males from the comparison group from this study had slightly higher proportions of being overweight to the population data from Switzerland (<24 years, n=5, 22% overweight), while the older males had lower portions (25-34 years, n=6, 23% overweight). None of the participants in the comparison group was classified as obese. The esports group had higher proportions of overweight individuals (<24 years, n=7, 23%; 25-34 years, n=9, 47%), but lower proportions of obese individuals (<24 years, n=1, 3%; 25-34 years, n=1, 5%) compared to the population data from Switzerland.

When comparing the results from this sample to previous laboratory-based esports studies which used the same objective measures, the esports players in this study had similar BMI average (mean 23.9, SD 3.1 kg/m^2^) compared with 23.7 (SD 3.3) kg/m^2^ [10] and 25.6 (SD 4.3) kg/m^2^ [43]. Based on BF%, this sample had an average of 18% (SD 5.6), which was just slightly higher than the results of Dykstra et al [43] of 16.5% (SD 8.3). The results of DiFrancisco-Donoghue et al [10] were much higher at 24.0% (SD 3.3) for their esports players. Both these studies had a similar mean age and demographic of esports players, and both recruited from university-level teams in the United States. Speculating about the discrepancy in weight status based on body fat percentage (BF%) is challenging. However, besides cultural, and geographical differences, one potential explanation could be the specific genre of esports titles played by the sample. While Dykstra et al [43] did not provide this information, DiFrancisco-Donoghue et al [10] revealed their esports participants were competitive in Overwatch (first-person shooter) as well as multiplayer web-based battle arenas video games. Notably, 12 (24%) of the current esports sample was competitive in sports genre video games. It is possible players who compete in sports genre video games may exhibit a greater inclination toward physical sports. More studies need to explore this question, as there are very few laboratory-based studies to compare results with, and all have the problem of relatively small sample sizes. Additionally, it must be mentioned that DiFrancisco-Donoghue et al [10] used dual-energy absorptiometry to determine BF%. It has been shown that bioelectrical impedance analysis underestimates the total body fat mass and overestimates fat free mass in healthy young adults compared to dual-energy absorptiometry [44].

### Hemodynamics

Regarding BP and PWV, the esports group did not have higher values putting them at no greater cardiovascular risk compared to the comparison group. A high BP is arguably the strongest directly measurable risk factor for the long-term development of the majority of CVDs [22].

Only 1 study was found that cross-sectionally examined SBP in a sample of 17 male esports players (age mean 20, SD 2 years) performed by Sousa et al [45]. Their group average values were 122 (SD 10) mm Hg for SBP, while the current sample had similar values of 121 (SD 8) mm Hg. No population reference data could be found for a similar age range of people in Switzerland, so data from Germany was compared due to the geographical closeness as well as cultural similarities between the countries. Among the general population of 18-29-year-old men in Germany, 69.2% are classified as optimal or normal, while 22.7% fall into the high normal category, and 7.9% are classified as having grade 1 hypertension [46]. When analyzing only the male participants from both groups in the current sample, a higher proportion of individuals reported optimal or normal BP values. Specifically, in the esports group, 43 (89%) participants fell into this category, while in the comparison group 44 (90%) participants had optimal or normal BP values. Based on this cross-sectional assessment of BP, esports players cannot be considered unhealthier or at a higher CVD risk compared to the comparison group or to a similar age demographic from the general German population.

The additional measurement of cardiovascular risk taken was PWV. Evidence suggests that PWV is more strongly associated with preclinical organ damage and is a better predictor of future cardiovascular events than peripheral BP [23]. According to data from prospective studies, a higher PWV is associated with a decline in endothelial function and is a precursor for future cardiovascular risk even after accounting for other established risk factors [47].

No existing studies have examined this variable among esports players, so no comparison of the results can be made in this regard. There were no differences between the groups regarding the PWV. Reference values provided from health centers across Europe reported mean values of 6.2 (SD 1.4) m/second for individuals less than 30 years [39]. Both groups from this study fall within the lower range of the European reference values suggesting relatively healthy values of arterial stiffness.

In summary, it appears competitive esports players have no increased risk of CVD compared to the age-matched comparison group or to a similar age demographic among the European population.

### Physical Fitness

Physical fitness, as measured by VO_2_max, was not statistically lower among the esports players in the sample. However, the esports players could be found more at the extreme ends of the classification spectrum for physical fitness compared to the comparison group.

Unfortunately, no previous study has directly assessed physical fitness among esports players. Only the VO_2_max values predicted based on a submaximal exercise test performed by Dykstra et al [43] were available for comparison. Compared to the predicted VO_2_max values from the esports sample of Dykstra et al [43], the current sample’s male esports average was comparable at 45.9 (SD 8.7) mL/kg/minute compared with 45.7 (SD 11.8) mL/kg/minute.

The relatively high percentage of esports players showing excellent or superior physical fitness was unexpected. These findings are particularly relevant as physical fitness is known to be a strong predictor of all-cause mortality [21]. Notably, previous studies have shown that individuals with high levels of physical fitness may mitigate the cardiovascular risks associated with a higher BMI [48,49]. This observation is consistent with our study on esports players who had a higher prevalence of being overweight and having obesity yet did not exhibit higher BP values.

One possible explanation for the high physical fitness level could be the high percentage of participation in sports genre video games. As speculated earlier, esports players competing in sports video games may have a vested interest or active participation in the respective sport outside of video games. However, only 7 of the 17 esports participants classified as having “excellent” or “superior” physical fitness were actively involved in sports genre video game titles.

Regarding the MGS, both groups revealed similar values. Unfortunately, no previous study has assessed MHG in esports players. According to MGS reference values for sex and age [50], the majority of the esports players (69%) and comparison group (73%) were above the 50th percentile. Both groups showed a similar percentage reaching values above the 90th percentile (esports players 18% and comparison group 20%) and under the 25th percentile (esports players 14% and comparison group 12%).

### Physical Activity Levels

The hypothesis that esports players would report lower amounts of weekly PA compared to the comparison group could not be confirmed. On average, the esports group and the comparison group scored similarly on the GLTPAQ. Regarding minutes of MVPA, the esports players reported performing more weekly minutes compared to the comparison group. However, this difference did not reach statistical significance. Interestingly, both groups showed higher percentages reaching the World Health Organization’s PA guidelines than the average reported for 18-29-year-old men in Germany (66.5%) [51].

Previous research on PA levels in esports players using self-report questionnaires has produced conflicting findings. While some studies have reported lower PA levels in esports players compared to the general population [16], others have found that esports players engage in PA at levels that are comparable to or higher than those of the general population [13,52]. These discrepancies could be attributed to geographical differences or variations in performance levels among esports players [16].

It is still hard to draw firm conclusions from this as it is possible that PA activity is overestimated among the responses. Based on the relatively high physical fitness results from this study, the weekly MVPA reports from the esports group do appear to make sense and suggest this is a relatively fit and active sample of esports players. Further research with more long-term objective PA behavior data would be very useful among this demographic.

### Limitations

A major limitation was the convenience sampling of the participants. Only 2 female esports participants were recruited. Although this is likely an underestimation of the true involvement of females in competitive esports, the female participant’s results were kept for analysis due to the male dominance of competitive esports participation globally. Furthermore, participants were recruited at Universities and Universities of Applied Sciences throughout Switzerland, thus, representing a fairly high academic background. The cross-sectional nature of the study was also a limitation of the study as esports player health may fluctuate throughout the year based on the tournament and competition schedules of the players. Unfortunately, BF% was only assessed via bioimpedance scale, and PA by self-report, which is not the gold standard for assessing body composition or PA.

Despite the limitations, this study did have some major strengths compared to previous research on esports players. The main strength was the inclusion of a diverse assortment of objective outcome measures related to health and fitness. Objective measures have hardly been used to research this demographic, and this study presents an opportunity for future research on esports players to expand on these results using similar measurements. Another strength was the addition of the age-matched comparison group to directly compare esports players to their peers. Although no strong conclusions can be drawn on the health and fitness status of all esports athletes as a whole, the results do add novel knowledge to the growing field of esports research and also raise some interesting questions for further research to expand upon.

### Conclusions

While the health narrative directed toward esports players has been mainly negative, this laboratory-based study indicated that esports players are not less healthy or fit compared to their peers. However, it seems that esports players are very heterogeneous and seem to span across the whole range of the fitness and health spectrum. Thus, the generalized statements of the esports athlete as an obese and unhealthy individual may need to be reconsidered. While this study cannot claim to represent the entirety of the competitive esports population, it does highlight the diversity of health and fitness which exists among esports players. This is probably due in part to the large spectrum of video game titles, leagues, training programs, and player statuses which are all encompassed by the singular term esports. As the results of this study hinted at, specific attention to the specific genre of esports participation may be necessary to target health-promotion training plans most efficiently.

## Abbreviations

BF%: body fat
BP: blood pressure
CVD: cardiovascular disease
GLTPAQ: Godin Leisure Time Physical Activity Questionnaire
MGS: maximal grip strength
MVPA: moderate to vigorous physical activity
PA: physical activity
PWV: pulse wave velocity
SBP: systolic blood pressure
VO_2_max: maximal oxygen consumption
WHtR: waist-to-height ratio

## Data Availability

All raw data are available on request to the corresponding author.
